# Implementation for Sustained Impact in Teleophthalmology (I-SITE): applying the *NIATx Model* for tailored implementation of diabetic retinopathy screening in primary care

**DOI:** 10.1186/s43058-021-00175-0

**Published:** 2021-07-06

**Authors:** Alejandra Torres Diaz, Loren J. Lock, Todd D. Molfenter, Jane E. Mahoney, Deanne Boss, Timothy D. Bjelland, Yao Liu

**Affiliations:** 1 Department of Ophthalmology and Visual Sciences, University of Wisconsin School of Medicine and Public Health, 2870 University Ave., Ste. 206, Madison, WI 53705 USA; 2 Department of Industrial and Systems Engineering, University of Wisconsin-Madison, Madison, WI USA; 3 Department of Medicine, University of Wisconsin School of Medicine and Public Health, Madison, WI USA; 4 Department of Family Medicine and Community Health, University of Wisconsin School of Medicine and Public Health, Madison, WI USA; 5 Mile Bluff Medical Center, Mauston, WI USA

**Keywords:** Retinal screening, Implementation intervention, Implementation development, Tailored implementation, *NIATx Model*, Systems engineering, Stakeholder engagement, Primary care, Telemedicine, Rural

## Abstract

**Background:**

Teleophthalmology provides evidence-based, telehealth diabetic retinopathy screening that is underused even when readily available in primary care clinics. There is an urgent need to increase teleophthalmology use in the US primary care clinics. In this study, we describe the development of a tailored teleophthalmology implementation program and report outcomes related to primary care provider (PCP) adoption.

**Methods:**

We applied the 5 principles and 10 steps of the *NIATx* healthcare process improvement model to develop and test I-SITE (Implementation for Sustained Impact in Teleophthalmology) in a rural, the US multi-payer health system. This implementation program allows patients and clinical stakeholders to systematically tailor teleophthalmology implementation to their local context. We aligned I-SITE components and implementation strategies to an updated ERIC (Expert Recommendations for Implementing Change) framework. We compared teleophthalmology adoption between PCPs who did or did not participate in various components of I-SITE. We surveyed PCPs and clinical staff to identify the strategies they believed to have the highest impact on teleophthalmology use.

**Results:**

To test I-SITE, we initiated a year-long series of 14 meetings with clinical stakeholders (n=22) and met quarterly with patient stakeholders (n=9) in 2017. Clinical and patient stakeholder groups had 90.9% and 88.9% participant retention at 1 year, respectively. The increase in teleophthalmology use was greater among PCPs participating in the I-SITE implementation team than among other PCPs (p < 0.006). The proportion of all PCPs who used the implementation strategy of electing diabetic eye screening for their annual performance-based financial incentive increased from 0% (n=0) at baseline to 56% (n=14) following I-SITE implementation (p = 0.004). PCPs and clinical staff reported the following implementation strategies as having the highest impact on teleophthalmology use: reminders to ask patients about diabetic eye screening during clinic visits, improving electronic health record (EHR) documentation, and patient outreach.

**Conclusions:**

We applied the *NIATx Model* to develop and test a teleophthalmology implementation program for tailored integration into primary care clinics*.* The *NIATx Model* provides a systematic approach to engaging key stakeholders for tailoring implementation of evidence-based telehealth interventions into their local context.

Contributions to the literature

- Few studies provide systematic guidance on the process for engaging providers and patients to tailor implementation of evidence-based interventions.
- We describe the development of a successful teleophthalmology implementation program based on the NIATx Model. This systems engineering model operationalizes a process for supporting primary care provider adoption and systematic, tailored implementation with stakeholder input.
- We align I-SITE components and implementation strategies to an updated ERIC (Expert Recommendations for Implementing Change) framework.
- We identify implementation strategies that clinical stakeholders believed to have a high impact on increasing teleophthalmology use for diabetic eye screening in primary care.

## Background

Telehealth has the potential to greatly increase access to clinical care, especially for populations with limited access to healthcare due to distance, time, and financial constraints [1–4]. Successful telehealth programs have been largely implemented in single-payer health systems both in the USA and abroad [5–8]. Single-payer health systems have been successful in implementing telehealth because they provide highly-coordinated care through a shared electronic health record system and have unique funding structures [6]. Yet, the majority of the US patients obtain care within multi-payer health systems, which face greater financial and logistical challenges to implementing complex telehealth programs [6]. Few studies in dissemination and implementation science provide a stepwise process for overcoming these barriers using stakeholder input to tailor integration of interventions into their local context [9, 10].

There is an urgent need to facilitate widespread adoption of telehealth in multi-payer health systems to improve detection of diabetic retinopathy. Diabetic retinopathy remains the leading cause of blindness in the USA largely due to lack of screening [11]. Blindness from diabetes is projected to continue increasing dramatically due to our rapidly rising diabetes population [11]. Teleophthalmology can greatly increase screening and reduce blindness from diabetes through early detection and treatment [5, 12, 13]. It is an evidence-based method for diabetic eye screening endorsed by the American Diabetes Association [14] in which patients obtain retinal photos using specialized cameras located conveniently in the primary care clinic. However, a 5-year randomized controlled trial found a lack of sustained effectiveness beyond 18 months when implemented in multi-payer primary care clinics [15]. Of the multiple patient and provider-related barriers to teleophthalmology use, [16–18] our work and that of others have identified that the major barrier is lack of teleophthalmology integration into primary care workflows.

Few clinical interventions have been proposed to increase teleophthalmology use, and the majority have focused on patient-level educational interventions [9, 19, 20]. We previously identified multiple patient and primary care provider (PCP) barriers and facilitators to teleophthalmology use in a rural, multi-payer health system through individual interviews [17]. Based on our results from that qualitative study, we postulated that health systems-level implementation strategies would be effective for increasing teleophthalmology use in the same rural health system. We identified the *NIATx Model* as a model that could increase teleophthalmology use by allowing patients and clinical stakeholders (e.g., PCPs, staff, and administrators) to systematically tailor teleophthalmology implementation to the unique needs and resources of their clinic. The *NIATx Model* is a healthcare process improvement model originally developed by systems engineers for behavioral health [21]. We adapted the *NIATx Model* to develop and test a teleophthalmology implementation program, I-SITE: Implementation for Sustained Impact in Teleophthalmology [22].

I-SITE allows patients and clinical stakeholders to systematically tailor teleophthalmology implementation to their local context. We previously reported patient-level clinical outcomes and found that I-SITE implementation at a single primary care clinic (Main) sustained a significant overall increase in teleophthalmology use and diabetic eye screening rates among all 5 primary care clinics within a rural multi-payer health system over 2 years [23]. Yearly teleophthalmology use increased following I-SITE implementation by more than 4-fold (from 41 to 196), with a greater increase at the Main (odds ratio [OR] 10.0) versus the other 4 Outreach clinics (OR 1.69). Overall, diabetic eye screening rates within the same health system were sustained at 36% above baseline at 2-years post-implementation.

In this work, we describe the development of I-SITE by applying the *NIATx Model* and assessed provider adoption from the same study. We aligned I-SITE components and implementation strategies with those from an updated ERIC framework [24]. We tested the hypothesis that teleophthalmology adoption would be higher among PCPs who did versus those who did not participate in various components of I-SITE. We also report implementation strategies that PCPs and clinical stakeholders believed to be the most impactful for increasing teleophthalmology use.

## Methods

### Research setting and design

We partnered with the Mile Bluff Medical Center, a rural US multi-payer health system, where a teleophthalmology program was established in 2015. Mile Bluff is a federally designated Rural Health Clinic located in Juneau County, WI, where the population is 83.5% rural and ranks in the lowest quartile of Wisconsin counties based on both health outcomes and socioeconomic determinants [25]. The teleophthalmology program was developed based on the 2011 American Telemedicine Association Telehealth Practice Recommendations for Diabetic Retinopathy [17, 26].

In this type of store-and-forward telemedicine program, PCPs place orders for patients to obtain teleophthalmology imaging at their convenience (usually same-day). A single-field, a 45-degree photograph of the disc and macula, along with an anterior segment photograph, is obtained for each eye using a Topcon NW400 non-mydriatic fundus camera (Topcon Medical Systems, Inc., Oakland, NJ, USA). The digital images are evaluated remotely by the University of Wisconsin eye specialists who send reports to the PCP and patient within 1 week. Expedited scheduling of a follow-up clinical exam with a local eye care provider is performed when further in-person care is needed. The teleophthalmology service was billed using CPT 92227 (remote imaging for detection of retinal disease) [27]. However, teleophthalmology was underutilized in the first 2 years of the program, prior to the introduction of I-SITE, with fewer than 20 patients imaged per quarter among approximately 2000 patients with diabetes.

We applied the *NIATx Model* to develop Implementation for Sustained Impact in Teleophthalmology (I-SITE) (free online toolkit available at: http://hipxchange.org/I-SITE) and tested I-SITE at Mile Bluff. We used a skilled primary care practice facilitator who had training in the *NIATx Model* to serve as the I-SITE Coach and recruited a local implementation team to develop and test implementation strategies. The I-SITE Coach was responsible for setting meeting agendas, preparing presentation materials, summarizing prior meeting minutes, facilitating discussions, and assigning tasks for next steps. The I-SITE implementation team was comprised of a mixed group of clinical stakeholders from Mile Bluff including PCPs, patient care staff, information technology/electronic health records (IT/EHR) staff, and clinic administrators recruited as described below. Our ideal size for the implementation team was proposed to be 8–12 members to allow for input from all team members. We also planned to recruited a patient stakeholder group of similar size to further refine patient-targeted implementation strategies.

**Fig. 1:**
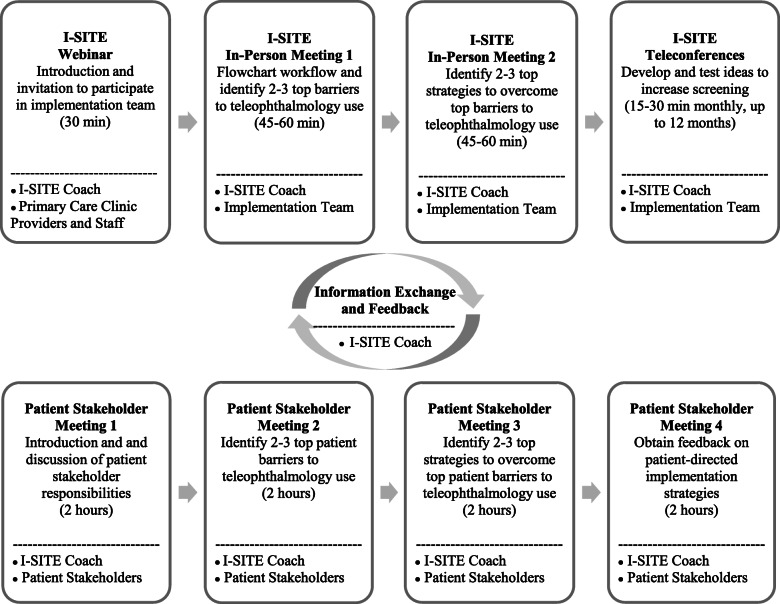
Implementation for Sustained Impact in Teleophthalmology (I-SITE) and patient stakeholder meeting goals and participants

The first 3 meetings of the implementation team were designed to be 1-h in length and conducted in-person to facilitate in-depth discussion regarding: (1) flowcharting the teleophthalmology workflow, (2) discussion of barriers to teleophthalmology use, and (3) strategies to overcome barriers to teleophthalmology use. Subsequent meetings were designed as a series of brief 15–30 min teleconferences to accommodate team members participating from different locations. These I-SITE in-person meetings and teleconferences incorporated the following components from the NIATx Model 10 steps: assemble the change team, plan the change, assign roles and tasks, and rapid cycle testing (Fig. 1). The teleconference meetings also encompass the following ERIC implementation strategies: implementation facilitation, organize implementation teams and team meetings, assess and redesign workflow, obtain feedback, use iterative strategies, and provide ongoing consultation, as well as audit and feedback. The Nominal Group Technique is used to generate ideas and to make decisions regarding the top barriers and strategies to test [17, 28]. With this technique, each individual on the implementation team sequentially offers one idea until no new ideas are generated. Each individual then votes for 2–3 ideas, after which the top 2–3 ideas endorsed by the group are identified through tallying the votes. The top strategies selected are then implemented by clinical providers and staff.

Given the busy clinical schedules of PCPs and clinical staff, it can be challenging to schedule meetings so we used teleconferences and regularly scheduled staff meetings whenever possible to facilitate participation. We also note that while the implementation team designed various strategies that were offered to all PCPs, the health system’s organizational culture empowered PCPs to independently select which strategies to implement in their clinical practice.

We measured I-SITE’s impact on teleophthalmology adoption by PCPs and surveyed PCPs and clinical staff to identify implementation strategies they perceived to have the greatest impact on increasing diabetic eye screening rates. The final intervention was reported according to guidance from TiDieR (Supplementary Material) [29].

### Patient and clinical stakeholder recruitment

Patients and clinical stakeholders were recruited in March 2017 from Mile Bluff to participate in two separate stakeholder groups to develop, test, and refine their teleophthalmology implementation with guidance from the I-SITE Coach. For our patient stakeholder group, we invited adult patients with diabetes who either had previous teleophthalmology imaging or had expressed interest in participating in research during a prior diabetic eye screening rate survey [17]. All patients were first mailed an invitation letter from the Chief Medical Officer at Mile Bluff, including the opportunity to opt-out. This was followed by a phone call from a member of our research team who provided a detailed description of the project and participant expectations. Patient stakeholder group participants received $50 for each meeting attended. For recruitment of our clinical stakeholder group, PCPs were provided with an introduction to the I-SITE program and invited to participate during an in-person presentation at a regularly scheduled staff meeting. Patient care staff who regularly worked with each participating PCP joined the clinical stakeholder team as well. Additional primary care staff and administrators (e.g., clinic management, quality improvement, and IT/EHR staff) were selected for participation by Mile Bluff leadership. No compensation was provided for clinical stakeholder group members as they were considered to be participating in quality improvement activities within the responsibilities of their employment.

### Outcome measures and statistical analysis

Data on teleophthalmology use was obtained using medical records abstraction. Patient and clinical stakeholder characteristics were obtained through self-report, medical records review, and review of personnel records at the Mile Bluff Medical Center. We used the Wilcoxon rank sum test to evaluate the change pre- and post-I-SITE implementation in teleophthalmology referrals among PCPs on the I-SITE implementation team versus PCPs not on the implementation team. We used McNemar’s test to assess the change in the number of PCPs who elected diabetic eye screening for their performance-based financial bonus each year. We also used t tests to compare yearly teleophthalmology referrals among PCPs who did versus PCPs who did not elect diabetic eye screening for their performance-based financial incentive. We identified implementation strategies perceived by PCPs and clinic staff to have the greatest impact on teleophthalmology use through a written survey conducted at a regularly scheduled staff meeting 2 years following I-SITE implementation, which was timed to align with our primary clinical effectiveness outcome reported previously [23]. All statistical analyses were performed using Microsoft® Excel (Microsoft Corp., Redmond, WA, USA).

### Ethics and institutional review board review

Our study was reviewed by the University of Wisconsin Health Sciences Institutional Review Board (IRB) staff and was determined to be exempt from full IRB review due the activities being consistent with providing quality improvement for implementing an evidence-based clinical practice within a health system. All research activities were conducted in accordance with the Declaration of Helsinki and all federal and state laws.

## Results

### Application of the NIATx Model in the development of Implementation for Sustained Impact in Teleophthalmology (I-SITE)

We applied the 5 principles and 10 steps of the *NIATx Model*, a systematic healthcare process improvement model, to develop I-SITE (Table 1). I-SITE allows for patients and clinical stakeholders to tailor teleophthalmology implementation through a series of meetings over the course of 1 year. These principles and steps from the *NIATx Model* and I-SITE align with several key implementation strategies selected from an updated ERIC framework (Table 1) [24]. The final version of I-SITE (Fig. 1) was developed as a modification of the process we applied in this study. The main modifications in the final version were that (1) the Introduction and Invitation to participate will be delivered as a live webinar during a regularly scheduled staff meeting for efficiency, rather than in-person, and that (2) the same implementation team, comprised of mixed group of clinical stakeholders, would gather for all I-SITE meetings. In this study, we had a larger clinical stakeholder group (n=22) present for the in-person I-SITE meetings, due to a greater-than-expected level of interest in participation. We subsequently met with a subset of this group (n=11) for implementation team teleconference meetings to achieve the desired group size to ensure input from all team members.

**Table 1:** Alignment between the ERIC framework [24] with *NIATx Model* and Implementation for Sustained Impact in Teleophthalmology (I-SITE)

		***NIATx Model*** 5 principles	Application in I-SITE
		1. Understand and involve the customer	Engage patients and clinical stakeholders (e.g. primary care providers, staff, and administrators) to understand and develop strategies to overcome barriers to teleophthalmology use
		2. Fix the key problems	Increase teleophthalmology use and diabetic eye screening rates in primary care clinics
**Selected Implementation Strategies from an** **Updated ERIC Framework** [24]		3. Pick a powerful Change Leader	Empower a clinic staff member who can be effective in obtaining buy-in from providers, staff and administrators at all levels
Implementation facilitation Assess for readiness and identify barriers and facilitators Conduct educational meetings Organize implementation teams and team meetings Assess and redesign workflow Obtain and use patients/consumers and family feedback Use evaluative and iterative strategies Provide ongoing consultation Audit and feedback		4. Get ideas from outside the organization/field	Borrow strategies from other areas of health maintenance (e.g. immunizations and dental appointment reminders)
		5. Use rapid cycle testing to establish effective changes	Test strategies cyclically with modifications made as needed to effectively increase teleophthalmology use and screening rates
		***NIATx Model*** **10 steps**	**Application in I-SITE**
		1. Identify a key problem	Explain the urgent need to increase teleophthalmology use and diabetic eye screening
		2. Do a walk-through	Walk-through the current teleophthalmology workflow
		3. Assemble the change team	Assemble an implementation team composed of clinical stakeholders (e.g. primary care providers, staff, and administrators) and a patient stakeholder team
		4. Flowchart	Flowchart the current teleophthalmology workflow with the implementation team to identify barriers to teleophthalmology use
		5. Plan the Change	Discuss potential strategies to increase teleophthalmology use and provide technical assistance
		6. Nominal Group Technique	Use this voting technique to reach a consensus on top barriers and top strategies to implement
		7. Assign Roles and Tasks	Assign roles and tasks to implementation team members to implement the selected strategies
		8. Rapid Cycle Testing	Test strategies, review data and feedback with the implementation team to decide whether to adopt, abandon, or adapt the changes across multiple short cycles
		9. Develop a Sustainability Plan	Select strategies that are highly likely to be sustainable or adapt strategies to improve sustainability
		10. Completion- Celebrate and Share Results	Share results with the primary care clinic, health system, and community at the end of the implementation period

### Patient and clinical stakeholder meetings

A total of 9 patients with diabetes and 22 clinical stakeholders participated in separate meetings from May 2017 to October 2018 to develop and iteratively test strategies to increase teleophthalmology use at Mile Bluff (Table 2). We held separate patient and clinical stakeholder groups due to differences in power dynamics, relationships, and terminology used among participants, which could limit patient stakeholder input. In addition, by dividing into two groups, we were able to include a greater total number of participants to lend their diverse perspectives. Information feedback and exchange between the two groups was facilitated by a practice facilitator (e.g., I-SITE Coach).

**Table 2:** Demographics of participants in Implementation for Sustained Impact in Teleophthalmology (I-SITE)

Patient stakeholders (n = 9)	Median (± SD) or percentage
Age	63.9 ± 8.1 years (range: 47–74)
Male	77.8%
Type II diabetes	100%
Experience with teleophthalmology	55.6%
Ethnicity	
White (non-Hispanic)	88.9%
White (Hispanic)	11.1%
Socioeconomic status	
Annual household income [30]	$48,117 ± $4115 (range: $37,396–$52,526)
Education	
Some high school	11.1%
High school graduate or GED	44.5%
Some college or technical school	22.2%
College graduate	22.2%
Health literacy (single-item literacy screener) [31]	
Low	22.2%
Moderate	55.6%
High	22.2%
**Clinical stakeholders (n = 22)**	**Percentage**
Male	13.7%
Clinical role	
Primary care provider (PCP)	36.4%
Physician (MD/DO)	22.7%
Physician assistant (PA-C)	4.5%
Nurse practitioner (APNP/DNP)	9.1%
Medical assistant (MA)	18.2%
Clinical administrator	22.7%
Diabetes educator	4.5%
IT/medical records	13.7%
Registration director	4.5%

The patient stakeholder group met four times approximately quarterly over a period of 1 year. Each patient stakeholder meeting was 2 h in length and held in the evenings with a light meal served beginning 30 min before the meeting. Each patient stakeholder meeting began with a community-building exercise (i.e., ice breaker question), followed by a brief review of the purpose and importance of obtaining the group’s input for increasing teleophthalmology use and diabetic eye screening in their community. Information was then presented on how patient stakeholder feedback from the last meeting was used to further the project’s goals. Meetings were facilitated by a research team member (Bachelor’s-level Research Specialist), and discussion topics included identifying top patient barriers and strategies for increasing teleophthalmology use and diabetic eye screening. In addition, patient stakeholder input was used to refine patient-targeted implementation strategies to increase teleophthalmology use, including patient education handouts, example scripts for PCPs and patient care staff to discuss teleophthalmology with patients, and the patient outreach process by which patients were reminded to obtain yearly teleophthalmology imaging. For patient outreach, reminder letters along with an enclosed patient education handout were mailed to patients who had obtained teleophthalmology 10 months previously to remind them to obtain their yearly eye screening. This was followed by a phone call to help assist with scheduling when needed.

The clinical stakeholder group was composed of 22 members who participated in three in-person meetings led by the I-SITE Coach. The first meeting of the clinical stakeholder group focused on creating a step-by-step flowchart of the primary care clinic workflow for identifying patients due for diabetic eye screening and placing orders for teleophthalmology. The second and third meetings employed the Nominal Group Technique to identify the top barriers to teleophthalmology use and the top implementation strategies based on those strategies participants expected to have the highest impact and be the most feasible to test within the next 2–3 weeks [17].

We subsequently brought together a subset of the clinical stakeholders to form a local implementation team to iteratively test implementation strategies. The implementation team (n = 11) consisted of 3 PCPs, 3 medical assistants (each whom works in the clinic with one of the 3 PCPs on the team), and 5 clinic administrative staff involved in quality improvement, IT/EHR support, and personnel management. This group met for a total of 11 meetings every 1–2 months for 15–30-min teleconferences led by the research team leader (Y.L.), a physician knowledgeable about primary care workflows, to review data and obtain feedback on implementation strategies. The I-SITE Coach did not lead these sessions in our study due to logistical reasons related to scheduling. The implementation team made decisions regarding whether to continue, abandon, or further refine each implementation strategy, as well as to propose new implementation strategies for testing.

We found that I-SITE had high retention of clinical and patient stakeholders at 12 months (90.9% (n=10) and 88.9% (n=9)), respectively). Patient stakeholders reported that they were motivated to continue participation because they received updates at each meeting about on how their feedback was used to make meaningful changes to the clinic’s implementation strategies. Other major factors patients reported for their continued participation included that they felt they were helping their community by contributing towards increasing diabetic eye screening. They also appreciated learning more about teleophthalmology and diabetic eye screening during these meetings, which allowed them to share that knowledge with family and friends with diabetes.

### Teleophthalmology adoption by primary care providers (PCPs)

The majority of PCPs (n=25) referred at least one patient for teleophthalmology in the year prior to and in the year following initiation of I-SITE implementation (n=18 (72.0%) and n=20 (80.0%), respectively). PCPs on the implementation team (n=3) had a greater increase in the number of teleophthalmology referrals pre- and post-I-SITE than other PCPs (n=22) (p < 0.006).

Of note, the PCP with the highest number of teleophthalmology referrals in the first year after initiation of I-SITE implementation was the first PCP to elect, on his own initiative, to tie his performance-based financial bonus to diabetic eye screening in that year (i.e., early adopter of this implementation strategy). This provider, who was on the implementation team, developed this implementation strategy during I-SITE implementation. In this health system, PCPs were able to choose to have their performance-based financial bonus tied to any major healthcare quality measure (e.g., hemoglobin A1c testing, mammography, colonoscopy) based on the proportion of their patients who had fulfilled that particular measure. We were unable to obtain details from the health system regarding the precise structure of this financial bonus as employee compensation is confidential.

In subsequent years, there was an increase in the number of PCPs who adopted this implementation strategy by electing diabetic eye screening as a performance-based financial incentive (up to 56%, n=14, of PCPs in 2020) (p = 0.004) (Table 3). PCPs who elected to have diabetic eye screening as a performance-based financial incentive referred more patients for teleophthalmology than those who did not make this election in 2018 and 2019 (p = 0.03 and p = 0.005, respectively). In 2020, we note that there was significant confounding related to the decline in teleophthalmology use beginning in quarter 2 due to reductions in primary care patient clinic visits at the Mile Bluff Medical Center related to safety concerns from the COVID-19 pandemic. In addition, the increase in overall referrals over time was not explained by an increase in eligible patients since the total numbers of patients with diabetes cared for by primary care providers at the Mile Bluff Medical Center was similar across all years of our study (average: 2293, range from 2119 to 2497).

**Table 3:** Teleophthalmology referrals among primary care providers (PCPs) based on election of diabetic eye screening performance-based financial incentive

	Year					
	2016	2017	2018	2019	2020	Average
**Proportion of PCPs electing incentive (n = 25)**	0 (0%)	1 (4%)	9 (36%)	4 (16%)	14 (56%)	5.6 (22%)
	**Total number of referrals**					
**All PCPs**	49	138	256	279	291	177.6
**PCPs who did elect incentive**	0	46	179	87	165	95.4
**PCPs who did not elect incentive**	49	92	77	192	126	107.2
***p*** **value**	N/A	N/A^a^	*p* = 0.03	*p* = 0.005	*p* = 0.11^b^	-

^a^The I-SITE intervention began in 2017. At that time, one PCP elected the incentive since no other PCPs were aware of this opportunity. Therefore, a t test was not used to compare referrals between PCPs who did and did not elect the incentive in 2017.

^b^2020 data were affected by the COVID-19 pandemic in that referrals declined in Spring 2020 as a result of reductions in primary care clinic visits due to safety concerns

### Teleophthalmology implementation strategies

The teleophthalmology implementation strategies tested are summarized in Table 4. All have been continued with the exception of the implementation team, which has been put on hold due to competing clinical demands related to the COVID-19 pandemic. We were not able to fully capture the duration or adaptation of every implementation strategy tested because the health system’s organizational culture empowered each PCPs to individually select some of the implementation strategies to use and when, as well as adapt the strategies and components of those strategies to meet each provider’s needs.

**Table 4:** Primary care provider (PCP) and clinical staff perceptions of implementation strategies’ impact on teleophthalmology use

Implementation strategy	Description	Updated ERIC framework [8]	High impact on teleophthalmology use^a^	
			PCPs (n=11) %	Clinical staff (n=14) %
Clinical reminders	Patient Rooming Checklist that reminds PCPs/Medical Assistants (MAs) to ask patients about last diabetic eye screening during a clinic appointment	Remind clinicians	**9 (81.8%)**	**11 (78.6%)**
Patient reminder phone calls	Yearly reminders (e.g., by mail, phone, or text message) to patients who had teleophthalmology performed previously and are due again for diabetic eye screening	Intervene with patients to enhance adherence	**8 (72.7%)**	**8 (57.1%)**
Improving EHR documentation	Consistent location of electronic health record (EHR) diabetic eye screening documentation	Change record systems	**7 (63.6%)**	**12 (85.7%)**
Patient education materials	Patient education materials (e.g., handouts, brochures, etc.) refined with patient stakeholder input	Develop educational materials	4 (36.4%)	**9 (64.3%)**
Provider and rtaff education	Presentations at regularly scheduled provider and staff meetings to provide education and updates on teleophthalmology use	Conduct educational meetings	2 (18.2%)	2 (14.3%)
Quarterly individual performance reports	Quarterly emails with individual provider-level data on the number of teleophthalmology referrals and comparison data with other providers	Audit and feedback	1 (9.1%)	0 (0.0%)
Implementation team	Monthly meetings with an implementation team of PCPs, MAs, and clinic administrators to develop implementation strategies and obtain ongoing feedback	Organize implementation teams and team meetings	0 (0.0%)	2 (14.3%)
Provider performance-based incentive	PCPs electing to have financial bonus for quality measure tied to their performance on diabetic eye screening rate among the PCP’s patient panel	Alter incentive structures	0 (0.0%)	0 (0.0%)
Bi-monthly group Performance reports	E-mail newsletters with updates on teleophthalmology program	Audit and feedback	0 (0.0%)	0 (0.0%)

^a^Endorsement by greater than 50% of respondents are highlighted in bold

However, we were able to obtain feedback on which strategies clinical stakeholders believed to have a high impact on teleophthalmology use using a written survey. The written survey was distributed to PCPs and clinical staff at regularly scheduled clinic meetings 2 years following initial I-SITE implementation, which was timed to align with our primary clinical effectiveness outcome that was reported previously [23]. At the time of the survey, there were a total of 25 PCPs and a total of 35 primary care clinical staff in the organization who would have been eligible to participate. At the meeting in which the survey was administered, there were 14 PCPs and 27 primary care clinical staff present who were invited to participate. Therefore, the response rate among those invited to participate was 78.6% (n=11) and 51.9% (n=14) among PCPs and clinical staff, respectively.

Strategies perceived by the majority of PCPs and clinical staff to have a high impact on increasing teleophthalmology use were reminders to ask patients about diabetic eye screening during clinic visits, improving the ease and consistency of diabetic eye screening documentation in the EHR (e.g., results from teleophthalmology imaging or from a dilated eye exam performed by the patient’s eye doctor), and outreach to contact patients due for diabetic eye screening (Table 4). Most clinical staff (n=9, 64.3%) believed that providing the patient education handout during a patient’s clinic visit had a high impact on increasing teleophthalmology use, but this strategy was endorsed by fewer PCPs (n=4, 36.4%).

Implementation strategies that were *not* considered to be high impact by the majority of PCPs and clinical staff included bi-monthly and quarterly emails with audit and feedback reporting of group- and individual-level data on teleophthalmology use (Table 4). PCP participation in the implementation team and PCP performance-based financial incentives also were not considered to be high impact by most clinical stakeholders. Notably, one PCP wrote in the word “No!” next to the survey item regarding whether financial incentives had a high impact. Only a minority of PCPs (n=2, 18.2%) and clinical staff (n=2, 14.3%) perceived teleophthalmology presentations providing education regarding the importance of teleophthalmology for diabetic screening and reporting yearly teleophthalmology use and diabetic eye screening rates at regularly scheduled provider and staff meetings to have a high impact on teleophthalmology use.

## Discussion

We adapted the *NIATx Model*, a healthcare process improvement model originally designed by systems engineers for behavioral health, to develop a systematic implementation program (I-SITE) to guide tailored teleophthalmology implementation in primary care clinics by patient and clinical stakeholders. We found that I-SITE increased provider teleophthalmology use, particularly among PCPs that participated in the I-SITE implementation team and among those that elected diabetic eye screening for their performance-based financial incentive. Data on strategies that increased provider teleophthalmology use complements our clinical outcomes demonstrating that I-SITE effectively sustained increased diabetic eye screening rates in a primary care-based teleophthalmology program in our prior work [23]. This study provides a model for adapting *NIATx* to guide implementation of evidence-based telehealth interventions to increase their adoption in primary care settings.

We aligned *NIATx Model* and I-SITE components to selected strategies from an updated ERIC framework developed by Perry et al. [24] based on ERIC utilization in a large-scale dissemination and implementation research program in primary care clinics. Among the suggested changes by these authors, they proposed an updated grouping of ERIC strategies as follows: (1) build health information technology to support data-informed quality improvement, (2) build quality improvement capacity and improve outcomes, (3) enhance clinician and practice member knowledge, and (4) build community connections and patient involvement [24]. Notably, I-SITE’s components and implementation strategies encompass all 4 of these categories, which may help explain why I-SITE was effective for increasing teleophthalmology use among PCPs. A team in Ireland also recently developed a proposed intervention to increase uptake of teleophthalmology diabetic eye screening by combining theory (i.e., Theoretical Domains Framework) with stakeholder engagement in a primary care setting [9]. All of the implementation strategies included in their final intervention are also operationalized within I-SITE, which was developed independently. According to Proctor et al.’s [32] recommendations for specifying implementation strategies, we note that the actors and target in I-SITE are primarily the practice facilitator and health system administrators (actors) working in concert to make systems-level changes to the clinical practice (target). While ERIC provides a valuable, standardized nomenclature for describing implementation strategies, the *NIATx Model* provides systematic guidance for the process by which implementation strategies are selected and utilized. The application of *NIATx* may be generalizable to integrating other telehealth interventions in primary care through tailoring implementation to fit the local context by key stakeholders.

The *NIATx Model* can be applied to engage local implementation teams across a broad range of settings and highlights the importance of practice facilitation. While *NIATx* was originally designed for increasing behavioral health services utilization, Pankow et al. [33] modified the *NIATx Model* to engage local implementation teams in correctional facilities to increase provision of HIV services. In this study, we successfully engaged patient and clinical stakeholders using I-SITE to sustain increased teleophthalmology use and diabetic eye screening rates by applying the *NIATx Model*. For our clinical stakeholder meetings, we invited individuals with varying roles within the organization to fill in critical information that may only be known to certain members of the organization, such medical assistants, EHR staff, and schedulers, to ensure that the meetings could move forward more effectively and efficiently. While sustaining engagement among clinical stakeholders may not always be practical in a primary care setting [34], we took advantage of previously scheduled meetings and used a teleconference format whenever possible to limit the burden of additional meetings. In addition, the *NIATx Model* provides a clearly defined role for a practice facilitator, which may be especially important for successful implementation within primary care clinics. A 2014 randomized controlled trial found that having a practice facilitator work in conjunction with a clinical process improvement team resulted in a significantly greater increase in traditional in-person diabetic eye screening, as well as other measures of diabetes care quality, compared to teams without a practice facilitator [35].

The Technology Acceptance Model, while not used in our work* a priori*, does however provide a useful model to help understand which implementation strategies clinical stakeholders identified to have the highest impact on teleophthalmology use. This model theorizes that two factors affect acceptance of technology: its perceived usefulness and perceived ease of use [36]. Rho et al. [37] found that both perceived usefulness and perceived ease of use were significantly associated with physicians’ intention to use telemedicine. Interestingly, implementation strategies identified by clinical stakeholders in our study as having the highest impact on teleophthalmology use were all related to increased perceived ease of use by clinical stakeholders (i.e., reminders to ask patients about diabetic eye screening during clinic visits, improving electronic health record (EHR) documentation, and patient outreach). In addition, we believe “perceived usefulness” was already high among PCPs since the implementation strategy of ‘Provider and Staff Education’ was considered by a minority of PCPs (n=2 (18.2%)) to have a high impact on teleophthalmology use. In addition, the majority of PCPs had referred patients for teleophthalmology prior to I-SITE and it would be highly unlikely for a busy PCP to take the time to explain, recommend, and order this type of test for a patient if he or she did not perceive that it would be useful. Implementation strategies targeting clinical stakeholders to increase teleophthalmology use in primary care may be most effective, therefore, if they primarily focus on improving perceived ease of use rather than perceived usefulness. Similar strategies may be effective for other types of evidence-based screening programs managed by primary care providers such as mammography, as well as for other store-and-forward telemedicine programs such as teledermatology.

Multiple studies have assessed the effectiveness of provider-based financial incentives to increase preventive screening. Levin-Scherz et al. [38] found that when physician bonuses were tied to quality measures, diabetic eye screening rates increased significantly. Similar results were found with increased hemoglobin A1c testing in a federally qualified health clinic where physicians could earn back withheld portions of their salaries by meeting certain diabetes quality measure targets [39]. However, a 2011 Cochrane review found insufficient evidence to either support or discourage the use of provider financial incentives to improve quality metrics in primary care [40]. Cultural attitudes may also lead providers and healthcare staff to ambivalently or negatively perceive the influence of provider financial incentives [41]. This may explain why few PCPs and clinical staff endorsed provider financial incentives as having a high impact on teleophthalmology use despite our data supporting its effectiveness in this study.

In addition, PCPs who volunteered to participate on the I-SITE implementation team referred significantly more patients for teleophthalmology than other PCPs following I-SITE implementation. PCPs on the implementation team were actively associated with supporting the intervention, a role defined by the Consolidated Framework for Implementation Research (CFIR )[42] as being a “champion.” Prior studies have demonstrated the importance of champions or peer leaders for increasing adoption of telehealth and evidence-based interventions at the organizational level, but have not examined the effect of being a champion on individual provider behavior [43, 44]. Of note, the provider champions in our study did not demonstrate higher teleophthalmology use *prior* to I-SITE implementation compared to other PCPs. It is possible that participation as a champion in the I-SITE implementation team resulted in a higher “dose” of provider engagement and buy-in that led to increased teleophthalmology use among these PCPs. We note that since participation on the implementation team was voluntary, these PCPs likely had individual characteristics (e.g., level of personal interest, self-efficacy, and motivation) that differed from other PCPs. Therefore, it seems unlikely that either random assignment or formal appointment of PCPs to the implementation team would have resulted in the same effect on an individual provider’s teleophthalmology use.

Limitations of our study included that we used a pre-post study design in a single rural health system with a small sample size of primary care providers serving a predominantly white, non-Hispanic population, as reflected in the demographics of our patient stakeholders (Table 2). Additional studies testing I-SITE across multiple health systems, including urban health systems and those serving non-white populations, are needed to further assess the generalizability of our findings. In addition, the health system’s organizational culture empowered each PCPs to individually select some of the implementation strategies to use and when. Therefore, multiple strategies were tested across overlapping time periods, which made it difficult to precisely quantify the relative contributions of each implementation strategy towards increasing teleophthalmology use, particularly for such strategies as patient rooming checklists (i.e., each PCP used their own, rather than a clinic-standardized, checklist), consistent documentation of diabetic eye screening in the EHR, patient outreach, as well as bimonthly and quarterly emailed performance reports. However, we did survey clinical stakeholders to ascertain their perceptions regarding high impact implementation strategies, with excellent response rates of 50–80% [45]. Finally, we did not ask patient stakeholders about which implementation strategies they perceived to have the highest impact on teleophthalmology use. The majority of tested strategies were directed at clinical stakeholders, rather than patients, based on our prior qualitative work that identified systems-based workflow strategies as being the most likely to be effective for increasing teleophthalmology use in rural primary care clinics [17].

## Conclusions

We applied the *NIATx Model* to develop and test a teleophthalmology implementation program that allows for tailored integration into primary care clinics. Our data suggest that the *NIATx Model* provides a systematic and effective approach to engaging key stakeholders for tailoring implementation of evidence-based telehealth interventions to fit their local context.

## Data Availability

The dataset supporting the conclusions of this article is available by request to those who obtain approval following the guidelines of the University of Wisconsin Health Sciences Institutional Review Board.

## Abbreviations

CFIR: Consolidated Framework for Implementation Research
EHR: Electronic health record
ERIC: Expert Recommendations for Implementing Change
I-SITE: Implementation for Sustained Impact in Teleophthalmology
IT: Information technology
*NIATx Model*: *NIATx Model* of healthcare improvement
PCP: Primary care provider
MA: Medical assistant
